# Retraction Note to: A new inducible transgenic mouse model for C9orf72-associated GGGGCC repeat expansion supports a gain-of-function mechanism in C9orf72-associated ALS and FTD

**DOI:** 10.1186/s40478-016-0401-9

**Published:** 2016-12-09

**Authors:** Renate K. Hukema, Fréderike W. Riemslagh, Shamiram Melhem, Herma C. van der Linde, Lies-Anne W. F. M. Severijnen, Dieter Edbauer, Alex Maas, Nicolas Charlet-Berguerand, Rob Willemsen, John C. van Swieten

**Affiliations:** 1 Department of Clinical Genetics, Erasmus Medical Center, 3015 CE Rotterdam, The Netherlands; 2 Department of Neurology, Erasmus Medical Center, 3015 CE Rotterdam, The Netherlands; 3 German Center for Neurodegenerative Diseases, 81337 Munich, Germany; 4 Department of Cell Biology, Erasmus Medical Center, 3015 CE Rotterdam, The Netherlands; 5 Department of Neurobiology and Genetics, IGBMC, INSERM U964, CNRS UMR7104, University of Strasbourg, Illkirch, France; 6 Department of Neurology, Neuroscience Campus Amsterdam, 1007 MB Amsterdam, The Netherlands; 7 PO Box 2040, 3000 CA Rotterdam, The Netherlands

## Retraction note

The authors are retracting this article [1]. Careful re-examination of the transgenic mice used in this study has indicated that they contain a transgenic sequence containing a 90CGG repeat, associated with fragile X-associated tremor/ataxia syndrome (FXTAS). Apparently, a mixture of two constructs containing the G4C2 repeat and the CGG repeat sequence was injected in oocytes to generate transgenic mice. The presence of the CGG repeat can explain the neuropathology described in the mice used for this study. We are therefore unable to present this transgenic mouse as model for C9orf72 related amyotrophic lateral sclerosis (ALS) and frontotemporal dementia (FTD).
